# A new species of cascade frog (Anura: Ranidae: *Amolops*) from central Yunnan, China

**DOI:** 10.1186/s40851-023-00214-9

**Published:** 2023-07-17

**Authors:** Shangjing Tang, Tao Sun, Shuo Liu, Sangdi Luo, Guohua Yu, Lina Du

**Affiliations:** 1 Key Laboratory of Ecology of Rare and Endangered Species and Environmental Protection, Guangxi Normal University, Ministry of Education, Guilin, 541004 China; 2 Guangxi Key Laboratory of Rare and Endangered Animal Ecology, College of Life Science, Guangxi Normal University, Guilin, 541004 China; 3 Kunming Natural History Museum of Zoology, Kunming Institute of Zoology, Chinese Academy of Sciences, Kunming, 650223 China

**Keywords:** *Amolops mantzourm*, Species group, The Qinghai-Tibetan Plateau, 16S rRNA, COI, Cytb

## Abstract

A new species of the genus *Amolops*, *Amolops ailao* sp. nov., is described from central Yunnan, China. The new species belongs to the *A. mantzorum* species group. Phylogenetic analyses based on the combination of mitochondrial 16S rRNA, COI, and cytb genes revealed that the new species is the sister taxon to *Amolops ottorum* with strong support. Genetically, the new species differs from *A. ottorum* by 5.0% in cytb sequences. Morphologically, the new species can be distinguished from known congeners by the combination of the following characters: true dorsolateral folds absent, but dorsolateral folds formed by series of glands present; circummarginal groove on tip of first finger absent; body size small (males SVL 33.0–35.1 mm and female SVL 41.3 mm); HW/SVL 0.32‒0.35; UEW/SVL 0.08‒0.10; THL/SVL 0.52‒0.56; vomerine teeth absent; interorbital distance narrower than internarial distance; tympanum distinct, less than half eye diameter; supratympanic fold present, indistinct; a pair of large tubercles on sides of cloaca; tibiotarsal articulation reaching beyond anterior corner of eye; and vocal sac absent. The cladogenesis events within the *A. mantzorum* group rapidly occurred from Pliocene 4.23 Mya to Pleistocene 1.2 Mya, coinciding with the recent intensive uplift of the Qinghai-Tibetan Plateau since the Pliocene. Combining findings in this study with the most recent taxonomic progress, we consider that there are 20 known *Amolops* species in Yunnan, China, accounting for the highest proportion of amphibian diversity of Yunnan, and five of them belong to the *A. mantzorum* group. Among different subfauna and water systems in Yunnan, the species diversity of *Amolops* in northwestern Yunnan and Nu River Basin is highest.

## Introduction

The cascade frogs of genus *Amolops* Cope, 1865 [1] inhabit rocky streams or waterfalls, enabled by abdominal suckers in larvae and enlarged digital discs in adults [2], and are widely distributed from Nepal and northern India eastwards to China and southwards to Malaysia [3]. The species diversity in *Amolops* has been poorly understood owing to morphological conservation [4, 5], and efforts relying on molecular data during the last decade have greatly improved our understanding of the taxonomy and species diversity of this genus, with a high number of new species having been discovered (e.g., [3, 4, 6–13]). So far, as the most speciose genus within the family Ranidae, the genus *Amolops* contains 79 species [14], which can be allocated 10 species groups [11]. In China, a total of 50 *Amolops* species have been recorded [15] and most of them have been assigned to eight species groups, namely *Amolops chayuensis* group, *Amolops daiyunensis* group, *Amolops hainanensis* group, *Amolops mantzorum* group, *Amolops monticola* group, *Amolops marmoratus* group, *Amolops viridimaculatus* group, and *Amolops ricketti* group, based on morphological and molecular evidence [4, 7, 9, 11, 16–24].

The *A. mantzorum* species group was defined based on the absence of true dorsolateral folds (not formed by incomplete series of glands), circummarginal groove on the tip of first finger, tarsal fold and tarsal glands absent, and nuptial pad present on first finger in males [11, 16, 17, 25]. It was comprised of 11 species [11, 24], namely *Amolops mantzorum* (David, 1872) [26], *Amolops granulosus* (Liu and Hu, 1961) [27], *Amolops loloensis* (Liu, 1950) [28], *Amolops lifanensis* (Liu, 1945) [29], *Amolops xinduqiao* Fei, Ye, Wang, and Jiang, 2017 [25], *Amolops jinjiangensis* Su, Yang, and Li, 1986 [30], *Amolops tuberodepressus* Liu and Yang, 2000 [31], *Amolops sangzhiensis* Qian, Xiang, Jiang, Yang, and Gui, 2023 [24], *Amolops shuichengicus* Lyu and Wang, 2019 [20], *Amolops ottorum* Pham, Sung, Pham, Le, Zieger, and Nguyen, 2019 [3], and *Amolops minutus* Orlov and Ho, 2007 [32]. Recently, *A. xinduqiao* was placed into synonymy of *A. mantzorum* as a subspecies [33]. Thus, currently the *A. mantzorum* species group contains 10 species, of which two (*A. ottorum* and *A. minutus*) are only known from northwestern Vietnam and seven are known from southwestern China [14].

Yunnan is located in southwestern China and harbors a rich amphibian fauna in terms of species count and endemism. It has been known that there are four members of the *A. mantzorum* group in Yunnan, i.e., *A. jinjiangensis*, *A. loloensis*, *A. mantzorum*, and *A. tuberodepressus* [15]. In recent years, a series of new species or new records of *Amolops* have been discovered intensively from southwestern China [2, 5, 7, 11, 18, 20, 22–25, 34–36], suggesting that species diversity of *Amolops* in the region still remains underestimated and probably more species would be found. During recent field surveys in central Yunnan, China, we collected seven specimens of an *Amolops* species that morphologically resemble some members of the *A. mantzorum* group in that they lack a circummarginal groove on tip of the first finger and have folds formed by incomplete series of glands along the dorsolateral junction of the body (hereafter dorsolateral glandular folds). Molecular and morphological comparison supported that these specimens differ from other members of the genus *Amolops*. Thus, we considered them to represent a new *Amolops* species.

## Materials and methods

### Sampling

**Fig. 1:**
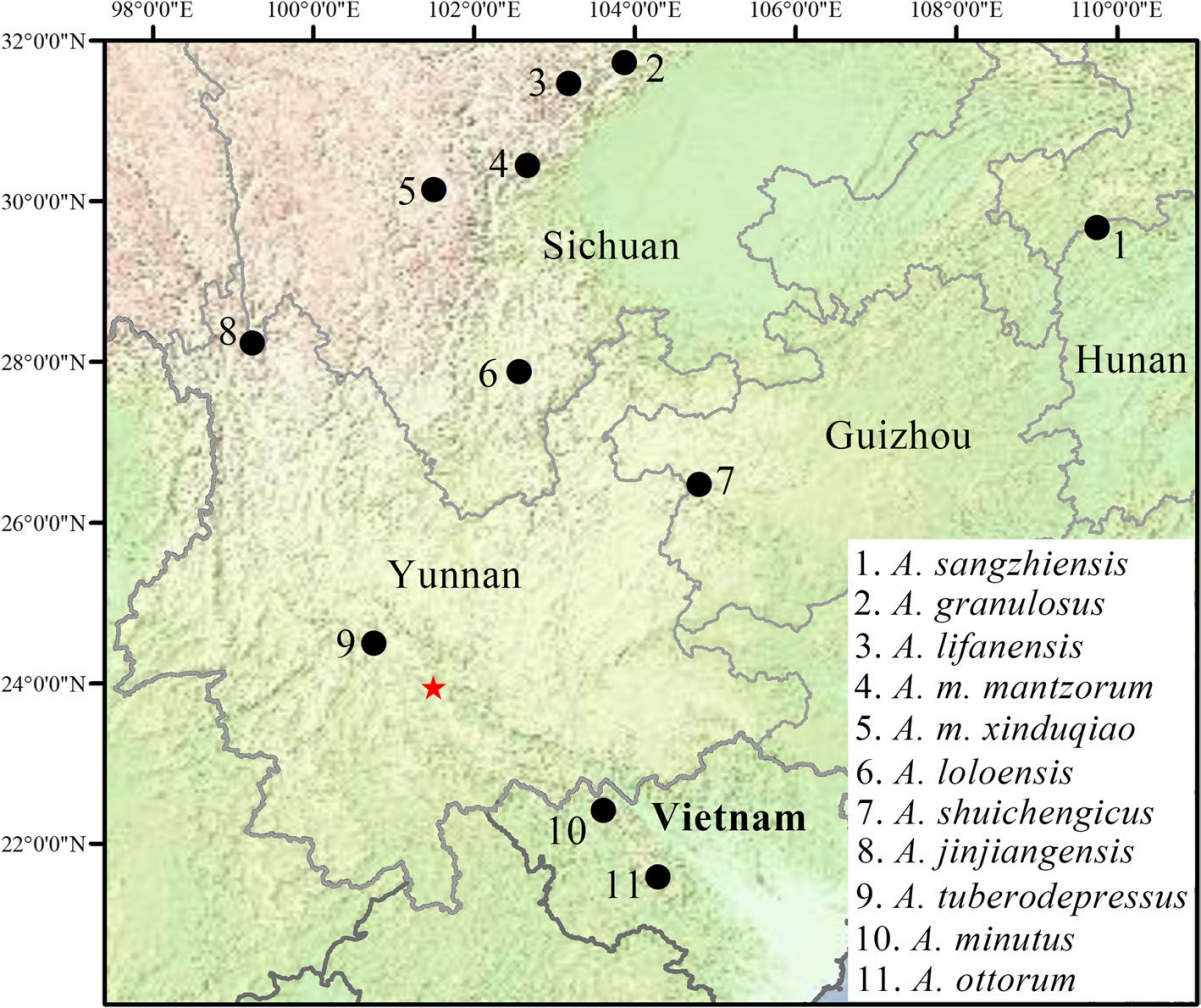
Map showing the collection site of *Amolops ailao* sp. nov. from central Yunnan, China (red star) and type localities of other known species and subspecies of *A. mantzorum* group (black circles)

Specimens were collected at Mt. Ailao, Xinping County, Yunnan Province, China (Fig. 1) by Guohua Yu in May 2019 and July 2019, and by Shuo Liu in June 2022. Specimens were photographed, euthanized, fixed, and then stored in 75% ethanol. Liver tissues were preserved in 99% ethanol. Specimens were deposited at Guangxi Normal University (GXNU) and Kunming Institute of Zoology, Chinese Academy of Sciences (KIZ).

### Morphology

Morphometric data were taken using digital calipers to the nearest 0.1 mm. Morphological terminologies follow Fei et al. [25]. Measurements included: snout-vent length (SVL, from tip of snout to vent); head length (HL, from tip of snout to rear of jaws); head width (HW, width of head at its widest point); snout length (SL, from tip of snout to anterior border of eye); internarial distance (IND, distance between nares); interorbital distance (IOD, minimum distance between upper eyelids); upper eyelid width (UEW, maximum width of upper eyelid); eye diameter (ED, diameter of exposed portion of eyeball); tympanum diameter (TD, the greater of tympanum vertical and horizontal diameters); forearm and hand length (FHL, from elbow to tip of third finger); thigh length (THL, from vent to knee); tibia length (TL, from knee to heel); foot length (FL, from proximal end of inner metatarsal tubercle to tip of fourth toe); length of foot and tarsus (TFL, from tibiotarsal joint to tip of fourth toe); and horizontal diameter of digital disc of third finger (F3DSC). Comparative morphological data of congeners were taken from their original descriptions or re-descriptions [2, 3, 6–13, 17–20, 22–25, 27–32, 34, 35, 37–59].

A multivariate principal component analysis (PCA) was conducted using SPSS 17.0 (SPSS Inc., USA) based on a correlation matrix of size-standardized measurements (all measurements divided by SVL). Scatter plots of the first two PCA factors were used to examine the differentiation between the new species and *A. ottorum*, which was recovered as the sister to the new species by phylogenetic analyses (see below). The measurements of *A. ottorum* were obtained from its original description [3].

### Molecular analyses

Total genomic DNA was extracted from liver tissues. Tissue samples were digested using proteinase K, and subsequently purified following a standard phenol/chloroform isolation and ethanol precipitation. Fragments encoding partial 16S rRNA (16S), partial cytochrome oxidase subunit I (COI), and partial cytochrome b (cytb) genes were amplified using primer pairs L2188 [60] /16H1 [61], Chmf4/Chmr4 [62], and F1/R3 [63], respectively. PCR amplifications were performed in 50 µl reactions using the following cycling conditions: an initial denaturing step at 95 °C for 4 min; 35 cycles of denaturing at 94 °C for 60s, annealing at 46, 49, or 51 °C for 60s (46 °C for COI, 49 °C for cytb, and 51 °C for 16S), and extending at 72 °C for 60s; and a final extending step of 72 °C for 10 min. Sequencing was conducted directly using the corresponding PCR primers. All new sequences have been deposited in GenBank under Accession Nos. MN650737–MN650749, MN650751‒MN650757, OP879227, OP880242, and OP887035 (Table 1). Homologous sequences of 25 congeners were obtained from GenBank. Two *Odorrana* species were included as outgroups according to Ngo et al. [64] and their sequences were also downloaded from GenBank.

**Table 1:** Species used for molecular analyses in this study

**Species**	**Voucher**	**Locality**	**16S**	**COI**	**CYTB**
*Odorrana wuchuanensis*	LBML 5230	Libo, Guizhou, China	KU680791	KU680791	KU680791
*Odorrana margaretae*	HNNU1207003	-	KJ815050	KJ815050	KJ815050
*Amolops afghanus*	SYS a003852	Tongbiguan, Yunnan, China	MG991895	MG991924	-
*Amolops bellulus*	-	-	DQ204473	KU243079	-
*Amolops chayuensis*	SYS a007509	Baxoi, Xizang, China	MK573820	MK568333	-
*Amolops chaoqin*	XM5526	Wenxian, Gansu, China	KX645666	KX645666	KX645666
*Amolops cremnobatus*	ROM 14528	Khe Moi, Nghe An, Vietnam	DQ204477	-	-
*Amolops daiyunensis*	SYS a001739	Mt. Daiyun, Fujian, China	MK263243	KX507328	-
*Amolops granulosus*	SYS a005316	Mt. Wawu, Sichuan, China	MK604851	MK605609	-
*Amolops granulosus*	20130258	Mt. Wawu, Sichuan, China	MH922934	MH922934	MH922934
*Amolops granulosus*	0700332	Anxian, Sichuan, China	-	-	KJ008439
*Amolops hainanensis*	SYS a005281	Mt. Wuzhi, Hainan, China	MK263281	MG991916	-
*Amolops hongkongensis*	DYTW-WYS-001	Mt. Wuyi, Fujian, China	KX233864	KX233864	KX233864
*Amolops jinjiangensis*	SYS a004571	Mt. Gaoligong, Yunnan, China	MK573801	MK568316	-
*Amolops jinjiangeniss*	SCUM050435CHX	Deqing, Yunnan, China	EF453741	MN961403	-
*Amolops jinjiangeniss*	CIB-XM6120	Benzilan, Deqing, Yunnan, China	MZ292455	MZ292455	MZ292455
*Amoops jinjiangensis*	KIZ047095	Chuxiong, Yunnan, China	MN953701	MN961404	-
*Amolops “jinjiangensis”*	IOZ4373	Zhongdian, Yunnan, China	-	-	KJ008379
*Amolops loloensis*	SYS a005351	Zhaojue, Sichuan, China	MK573806	MK568321	-
*Amolops loloensis*	SM-ZDTW-01	Shimian, Sichuan, China	KT750963	KT750963	KT750963
*Amolops loloensis*	0700211	Huanyuan, Sichuan, China	-	-	KJ008427
*Amolops tuberodepressus*	CIB-XM3125	Jingdong, Yunnan, China	KR559270	KR559270	KR559270
*Amolops tuberodepressus*	YU20160272	Mt. Ailao, Xinping, Yunnan, China	MN650757	-	MN650749
*Amolops tuberodepressus*	SYS a003931	Mt. Wuliang, Jingdong, Yunnan, China	MG991904	MG991933	-
*Amolops lifanensis*	SYS a005378	Lixian, Sichuan, China	MK604870	MK605628	-
*Amolops lifanensis*	0700039	Maoxian, Sichuan, China	-	-	KJ008445
*Amolops medogensis*	SYS a006657	Medog, Xizang, China	MK573813	MK568328	-
*Amolops mantzorum mantzorum*	-	Mt. Xiling Snow, Dayi, Sichuan, China	KJ546429	KJ546429	KJ546429
*Amolops mantzorum mantzorum*	SYS a005362	Fengtongzhai NR, Baoxing, Sichuan, China	MG991893	MG991922	-
*Amolops mantzorum mantzorum*	0700114	Longdong, Baoxing, Sichuan, China	-	-	KJ008297
*Amolops mantzorum mantzorum*	0700229	Chongzhou, Sichuan, China	-	-	KJ008339
*Amolops mantzorum* ssp.	0700040	Maoxian, Sichuan, China	-	-	KJ008277
*Amolops mantzorum* ssp.	0700267	Wenxian, Gansu, China	-	-	KJ008360
*Amolops mantzorum xinduqiao*	LCLH017	Luhuo, Sichuan, China	-	-	KJ008392
*Amolops mantzorum xinduqiao*	0700307	Yajiang, Sichuan, China	-	-	KJ008410
*Amolops mantzorum xinduqiao*	CIB-999	Kangding, Sichuan, China	-	-	KJ008415
*Amolops marmoratus*	CAS240593	Mon, Myanmar	JF794456	-	-
*Amolops marmoratus*	KUHE19089	Chiang Mai, Thailand	AB211486	-	AB259738
*Amolops ottorum*	IEBR 4342	Muong La, Son La, Vietnam	-	-	MK941135
*Amolops ottorum*	TBU 06	Muong La, Son La, Vietnam	-	-	MK941136
*Amolops viridimaculatus*	SYS a003813	Mt. Gaoligong, Yunnan, China	MK604836	MK605597	KJ008459
*Amolops torrentis*	SYS a005289	Mt. Wuzhi, Hainan, China	MK263284	MG991930	-
*Amolops sangzhiensis*	CSUFT905	Mt. Doupeng, Hunan, China	OQ079539	OQ078904	-
*Amolops sangzhiensis*	CSUFT907	Mt. Doupeng, Hunan, China	OQ079540	OQ078905	-
*Amolops shuichengicus*	SYS a004956	Shuicheng, Guizhou, China	MK604845	MK605603	-
*Amolops shuichengicus*	SYS a004957	Shuicheng, Guizhou, China	MK604846	MK605604	-
*Amolops spinapectoralis*	ROM 7513	Tram Lap, Gia Lai, Vietnam	DQ204487	-	-
*Amolops ricketti*	WUSTW01	Mt. Wugong, Jiangxi, China	KF956111	KF956111	KF956111
*Amolops wuyiensis*	-	Mt. Wuyi, Fujian, China	KJ933509	KJ933509	KJ933509
*Amolops ailao* sp. nov.	GXNU YU000001	Mt. Ailao, Xinping, Yunnan, China	MN650751	MN650737	MN650743
*Amolops ailao* sp. nov.	GXNU YU000002	Mt. Ailao, Xinping, Yunnan, China	MN650752	MN650738	MN650744
*Amolops ailao* sp. nov.	GXNU YU000003	Mt. Ailao, Xinping, Yunnan, China	MN650753	MN650739	MN650745
*Amolops ailao* sp. nov.	GXNU YU000004	Mt. Ailao, Xinping, Yunnan, China	MN650754	MN650740	MN650746
*Amolops ailao* sp. nov.	GXNU YU20160273	Mt. Ailao, Xinping, Yunnan, China	MN650755	MN650741	MN650747
*Amolops ailao* sp. nov.	GXNU YU20160274	Mt. Ailao, Xinping, Yunnan, China	MN650756	MN650742	MN650748
*Amolops ailao* sp. nov.	KIZ 2022041	Mt. Ailao, Xinping, Yunnan, China	OP879227	OP880242	OP887035

Sequences were aligned using MUSCLE with the default parameters in MEGA 7 [65]. Uncorrected pairwise distances between species were calculated in MEGA 7. Because sequences of the three genes are not all available for each species (Table 1), we prepared a combined dataset of the three genes for phylogenetic analyses. The best substitution model was selected using the Corrected Akaike Information Criterion (AICc) in jMODELTEST v. 2.1.6 [66] via Cipres Science Gateway [67]. Bayesian inference was performed in MRBAYES 3.2.6 [68] based on the selected substitution model (GTR + I + G). Two runs were performed simultaneously with four Markov chains starting from random tree. The chains were run for 3,000,000 generations and sampled every 100 generations. Burn-in was checked using the program Tracer v.1.6 [69]. The first 25% of the sampled trees were discarded as burn-in and then the remaining trees were used to create a consensus tree and to estimate Bayesian posterior probabilities (BPPs). In addition, a maximum likelihood (ML) analysis was conducted in RAXML-HPC v.8.2.12 [70] with 1000 rapid bootstrap replicates.

We estimated the lineage divergence times using an uncorrelated lognormal relaxed molecular clock model in BEAST v. 1.8.0 [71]. The birth-death process was chosen as the tree prior because of the mixed inter- and intraspecies sampling in the dataset sets [72]. Recently, the crown age for *Amolops* was inferred to be 25.01 Ma (95% HPD: 21.31‒28.58) by Wu et al. [4]. Therefore, the time of most recent common ancestor (TMRCA) of the genus *Amolops* (25.01 ± 2.2 Ma) was used as a secondary calibration point based on Wu et al. [4]. A run of 20 million generations was conducted by sampling every 1000 generations. The effective sample sizes of values of parameters were examined using Tracer v.1.6 [73]. The maximum clade credibility tree was constructed in TreeAnnotator v.1.8.0 [71] and was visualized in FigTree v.1.4.0 (from http://tree.bio.ed.ac.uk/software/figtree).

## Results

### Phylogeny and divergence time estimation

**Fig. 2:**
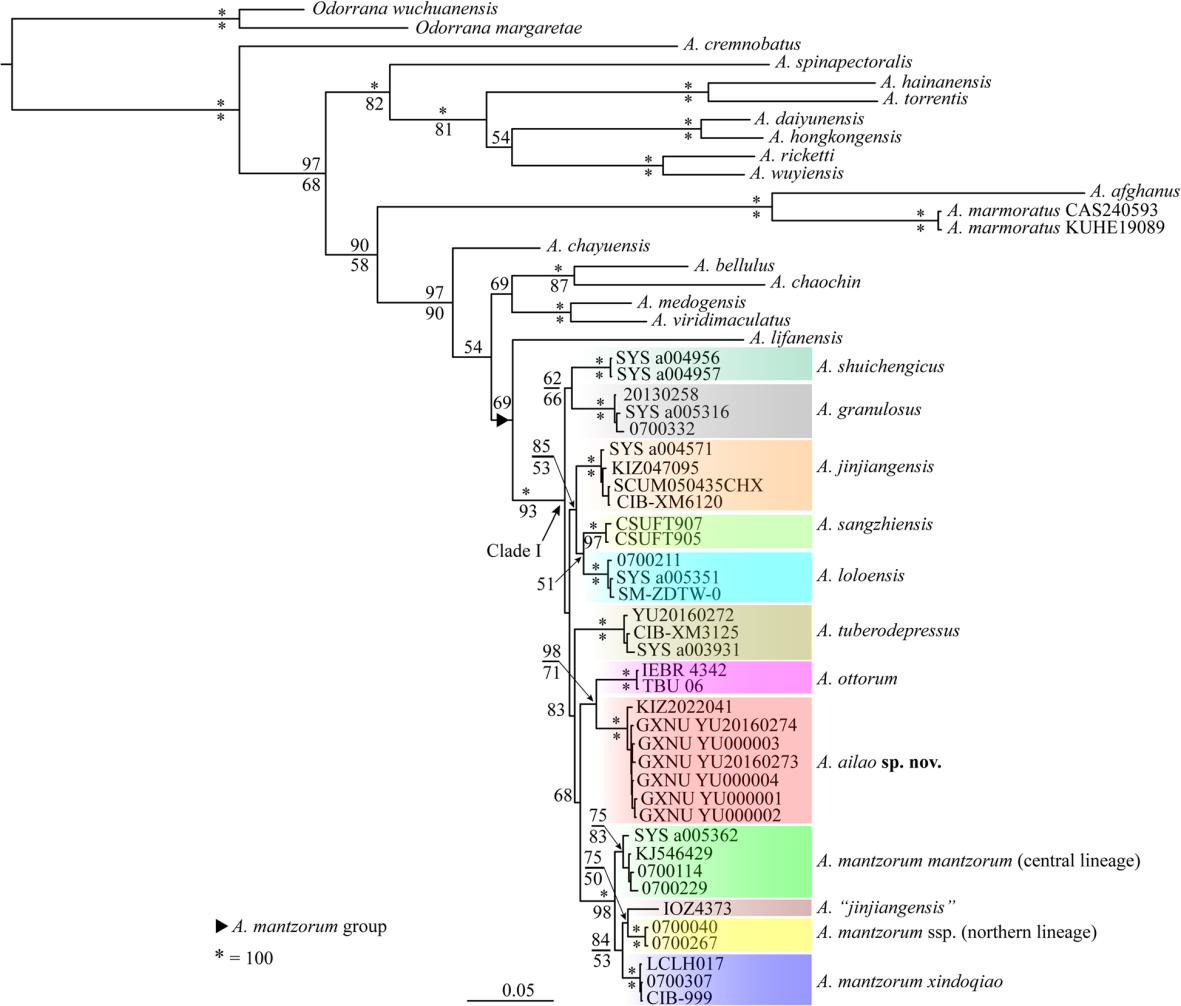
Bayesian phylogram of *Amolops* inferred from the combination of 16S rRNA, COI and cytb sequences. Numbers above and below branches are Bayesian posterior probabilities and ML bootstrap values (only values above 50% are shown), respectively

Alignments of 16S, COI, and cytb genes were 884 bp, 676 bp, and 998 bp in length, respectively. The newly collected individuals from Mt. Ailao, Yunnan, China represented a distinct lineage nested in the clade of members of the *A. mantzorum* group, and it was recovered as the sister taxon to *A. ottorum* with strong supports (Fig. 2). Genetic distances (p-distance) between this lineage and other species in the *A. mantzorum* group ranged from 1.9% (vs. *A. sangzhiensis*) to 8.3% (vs. *A. lifanensis*) in 16S (Table 2), from 4.0% (vs. *A. mantzorum mantzorum*) to 14.2% (vs. *A. lifanensis*) in COI (Table 2), and from 5.0% (vs. *A. ottorum*) to 14.4% (vs. *A. lifanensis*) in cytb (Table 3). The specimen IOZ4373, which was identified as *A. jinjiangensis* in Lu et al. [63], was not clustered together with topotypes of *A. jinjiangensis* (Fig. 2).

**Table 2:** Genetic distance (%) between members of the *A. mantzorum* group estimated from 16S (lower triangle) and COI sequences (upper triangle). *A. m. mantzorum* = *A. mantzorum mantzorum*; *A. m.* ssp. = *A. mantzorum* ssp.; *A. m. xinduqiao* = *A. mantzorum xinduqiao*

	1	2	3	4	5	6	7	8	9	10	11	12	13
1 *A. ailao* sp. nov.	‒	NA	4.7	5.8	4.0	NA	NA	NA	5.5	5.4	4.7	14.2	4.5
2 *A. ottorum*	NA	‒	NA	NA	NA	NA	NA	NA	NA	NA	NA	NA	NA
3 *A. loloensis*	2.1	NA	‒	3.1	3.5	NA	NA	NA	5.3	5.1	4.6	12.6	3.0
4 *A. jinjiangensis*	2.1	NA	1.0	‒	4.6	NA	NA	NA	5.7	5.2	4.9	13.7	4.7
5 *A. m. mantzorum*	2.2	NA	2.0	2.1	‒	NA	NA	NA	5.1	4.9	4.6	12.8	3.9
6 *A. m.* ssp.	NA	NA	NA	NA	NA	‒	NA	NA	NA	NA	NA	NA	NA
7 *A. “jinjiangensis”*	NA	NA	NA	NA	NA	NA	‒	NA	NA	NA	NA	NA	NA
8 *A. m. xinduqiao*	NA	NA	NA	NA	NA	NA	NA	‒	NA	NA	NA	NA	NA
9 *A. tuberodepressus*	2.7	NA	1.7	1.8	2.1	NA	NA	NA	‒	6.0	5.2	13.8	5.9
10 *A. granulosus*	2.7	NA	2.0	2.2	2.7	NA	NA	NA	2.2	‒	4.1	13.0	5.8
11 *A. shuichengicus*	3.0	NA	2.1	2.0	3.4	NA	NA	NA	3.0	2.9	‒	12.4	5.5
12 *A. lifanensis*	8.3	NA	7.6	7.5	9.0	NA	NA	NA	8.3	7.8	8.3	‒	12.4
13 *A. sangzhiensis*	1.9	NA	1.0	0.8	2.0	NA	NA	NA	1.9	2.1	2.0	8.0	

**Table 3:** Genetic distance (%) between members of the *A. mantzorum* group estimated from cytb sequences. *A. m. mantzorum* = *A. mantzorum mantzorum*; *A. m.* ssp. = *A. mantzorum* ssp.; *A. m. xinduqiao* = *A. mantzorum xinduqiao*

	1	2	3	4	5	6	7	8	9	10	11
1 *A. ailao* sp. nov.	‒										
2 *A. ottorum*	5.0	‒									
3 *A. jinjiangensis*	6.4	5.1	‒								
4 *A. m. xinduqiao*	6.7	6.5	6.1	‒							
5 *A. tuberodepressus*	6.7	5.9	5.8	6.8	‒						
6 *A. m.* ssp.	7.0	7.0	6.6	2.4	7.1	‒					
7 *A. m. mantzorum*	7.0	7.6	6.7	2.5	7.1	2.9	‒				
8 *A. “jinjiangensis”*	7.3	7.0	6.9	3.4	6.7	3.1	3.2	‒			
9 *A. loloensis*	7.0	5.9	5.1	5.8	6.5	7.1	6.8	7.0	‒		
10 *A. granulosus*	7.8	6.7	5.6	6.7	7.1	6.9	6.6	6.9	6.0	‒	
11 *A. lifanensis*	14.4	14.8	14.6	14.2	14.8	14.5	13.8	15.0	14.4	13.6	‒
12 *A. shuichengicus*	NA	NA	NA	NA	NA	NA	NA	NA	NA	NA	NA

**Fig. 3:**
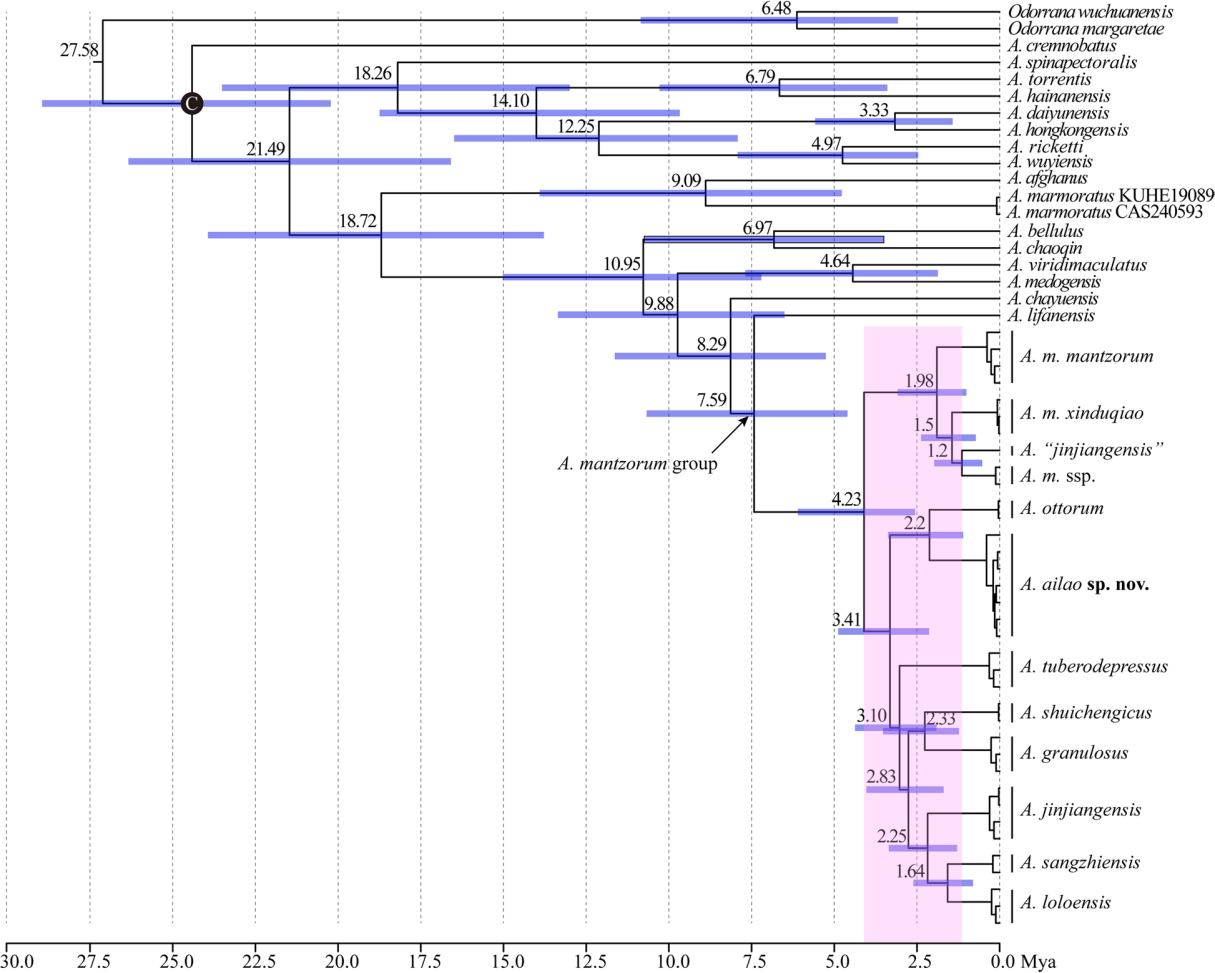
Divergence time estimates within *Amolops* using BEAST. Numbers above branches are average ages and blue bars represent 95% intervals. The calibration point is highlighted with a circle

The dating analysis revealed that *A. lifanensis* split from all other members of the *A. mantzorum* species group ca. 7.59 Mya (95% HPD: 4.6‒10.67 Mya) and then the lineage divergence within the *A. mantzorum* species group mainly occurred from 4.23 Mya (95% HPD: 2.56‒6.1 Mya) to 1.2 Mya (95% HPD: 0.53‒1.98 Mya) (Fig. 3). The divergence between the novel lineage from Mt. Ailao and *A. ottorum* occurred 2.2 Mya (95% HPD: 1.1‒3.37 Mya).

### Morphometric analysis

**Fig. 4:**
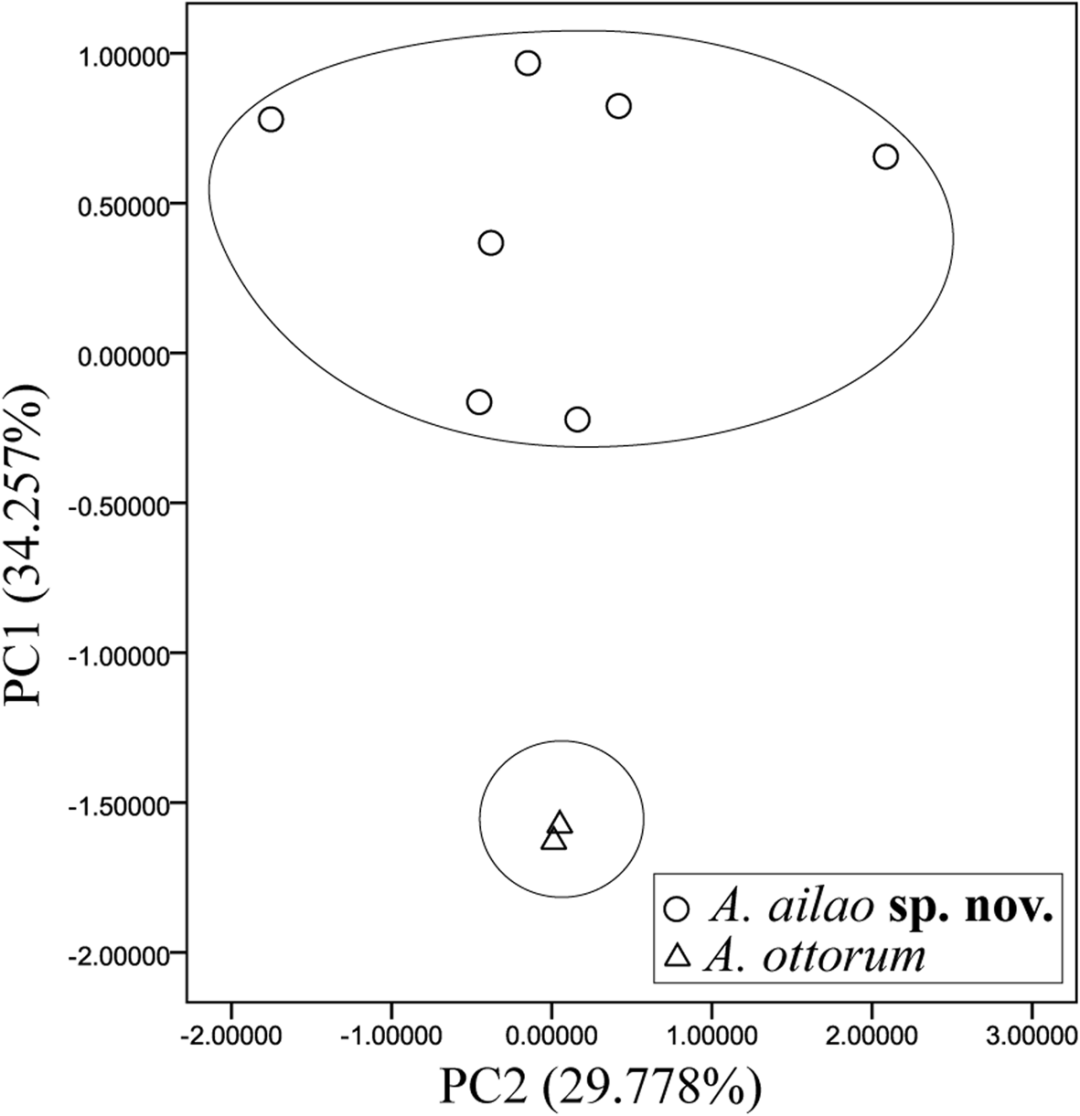
Scatterplot of principal components 1 and 2 of size-adjusted morphometric data for *A. ailao* sp. nov. and *A. ottorum*

Morphological measurements are given in Table 4. We retained the first two principal components, which had eigenvalues above 1.0 and accounted for 64.035% of total variance (Table 5). Loadings for PC1, which accounted for 34.26% of the total variance, were most heavily loaded on HW, UEW, ED, and THL (load factor > 0.7), and differentiation was found along the PC1 axis between the new species and *A. ottorum* (Fig. 4), indicating that the new species differs from *A. ottorum* by wider head, wider upper eyelid, larger eye, and longer femur. The second principal component (PC2) accounted for 29.78% of the total variance, but no clear separation was observed along this axis between the new species and *A. ottorum*. In addition, the new lineage can be distinguishable from its congeners by body size and the combination of texture and coloration pattern.

**Table 4:** Measurements (mm) of the holotype and paratypes of *Amolops ailao* sp. nov. Abbreviations defined in the text

Character	GXNU YU000001	GXNU YU000002	GXNU YU000003	GXNU YU000004	GXNU YU20160273	GXNU YU20160274	KIZ 2022041
Sex	♂	♂	♂	♂	♂	♂	**♀**
SVL	33.0	35.1	33.4	33.6	33.7	33.4	41.3
HL	11.2	11.6	11.3	11.0	11.1	10.7	14.7
HW	10.8	11.4	11.1	10.8	11.0	10.6	14.4
SL	4.6	4.9	4.8	4.8	4.5	4.9	6.2
IND	3.5	3.9	3.7	3.7	3.7	3.6	5.0
IOD	3.2	3.3	3.1	3.3	3.1	3.1	3.4
UEW	3.2	2.8	3.3	3.0	3.3	3.0	3.6
ED	4.5	4.4	4.6	4.3	4.5	4.3	5.0
TD	1.6	1.6	1.6	1.7	1.6	1.4	1.9
FHL	17.5	18.5	17.1	17.6	17.2	17.3	22.0
THL	18.5	18.9	18.6	18.4	17.6	18.3	22.2
TL	19.2	20.0	19.1	20.1	18.7	19.1	23.2
TFL	26.7	29.1	26.9	29.5	26.7	27.0	32.8
FL	18.3	20.1	18.5	19.8	18.4	18.6	22.0
F3DSC	1.8	2.0	1.8	1.8	1.8	1.6	2.1

**Table 5:** Factor loadings of first two principal components of 12 size-adjusted morphometric characteristics of *Amolops ailao* sp. nov. and *A. ottorum*

Character	PC1	PC2
Eigenvalue	4.111	3.573
% variation	34.257%	29.778%
HW (head width)	**0.808**	-0.474
HL (head length)	0.566	-0.486
SL (snout length)	-0.097	-0.320
IND (internarial distance)	0.258	0.774
IOD (interorbital distance)	-0.469	0.787
UEW (width of upper eyelid)	**0.914**	-0.009
ED (eye diameter)	**0.704**	0.198
TD (tympanum diameter)	0.691	0.528
THL (thigh length)	**0.893**	0.121
TL (tibia length)	0.026	0.850
TFL (length of foot and tarsus)	0.375	0.767
F3SDC3 (diameter of disc of finger III)	-0.308	0.385

### Taxonomic account

**Fig. 5:**
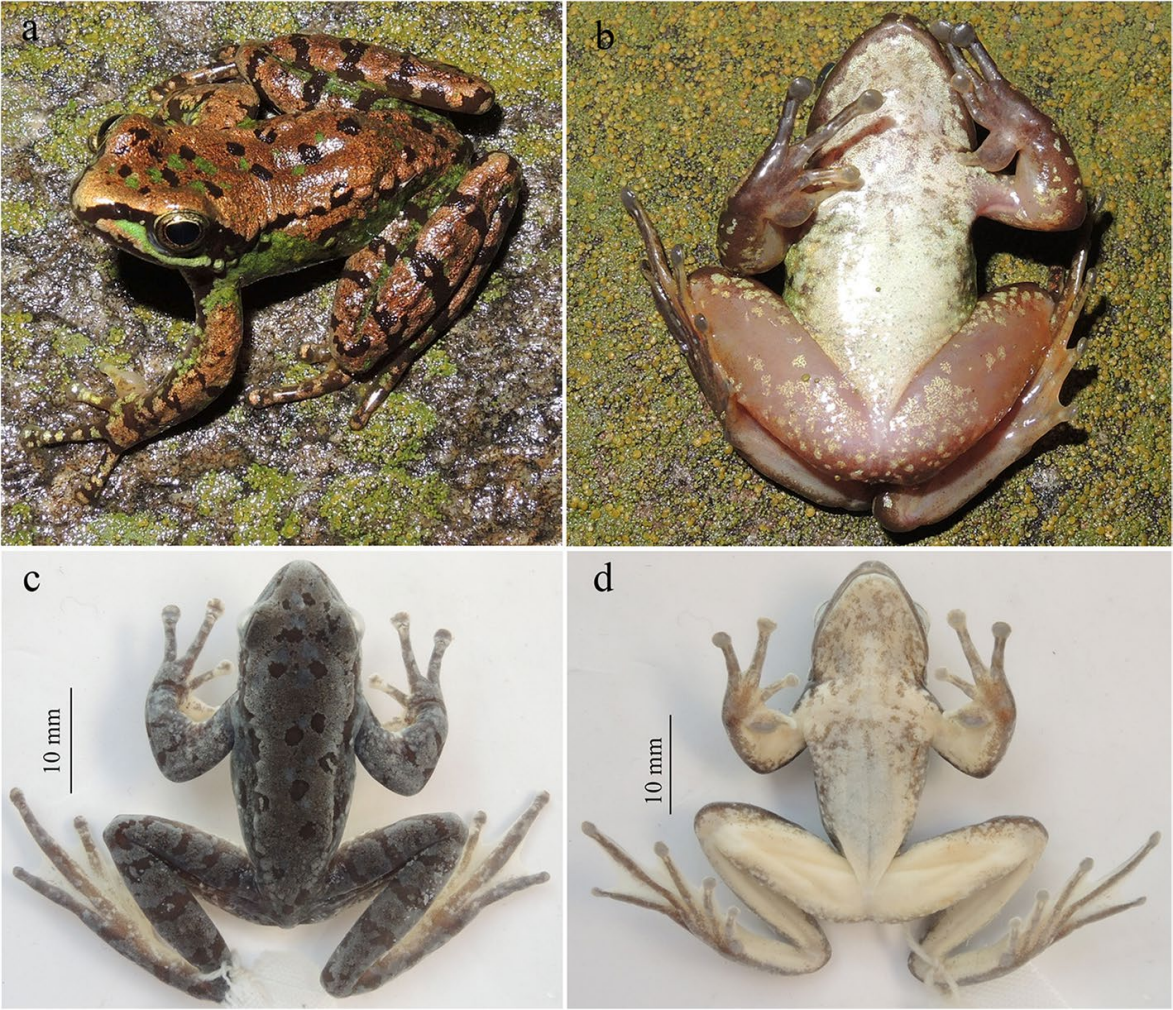
Holotype of *Amolops ailao* sp. nov. in life (**a** and **b**) and in preservative (**c** and **d**). (**a**) dorsolateral view, (**b**) ventral view, (**c**) dorsal view, and (**d**) ventral view

**Fig. 6:**
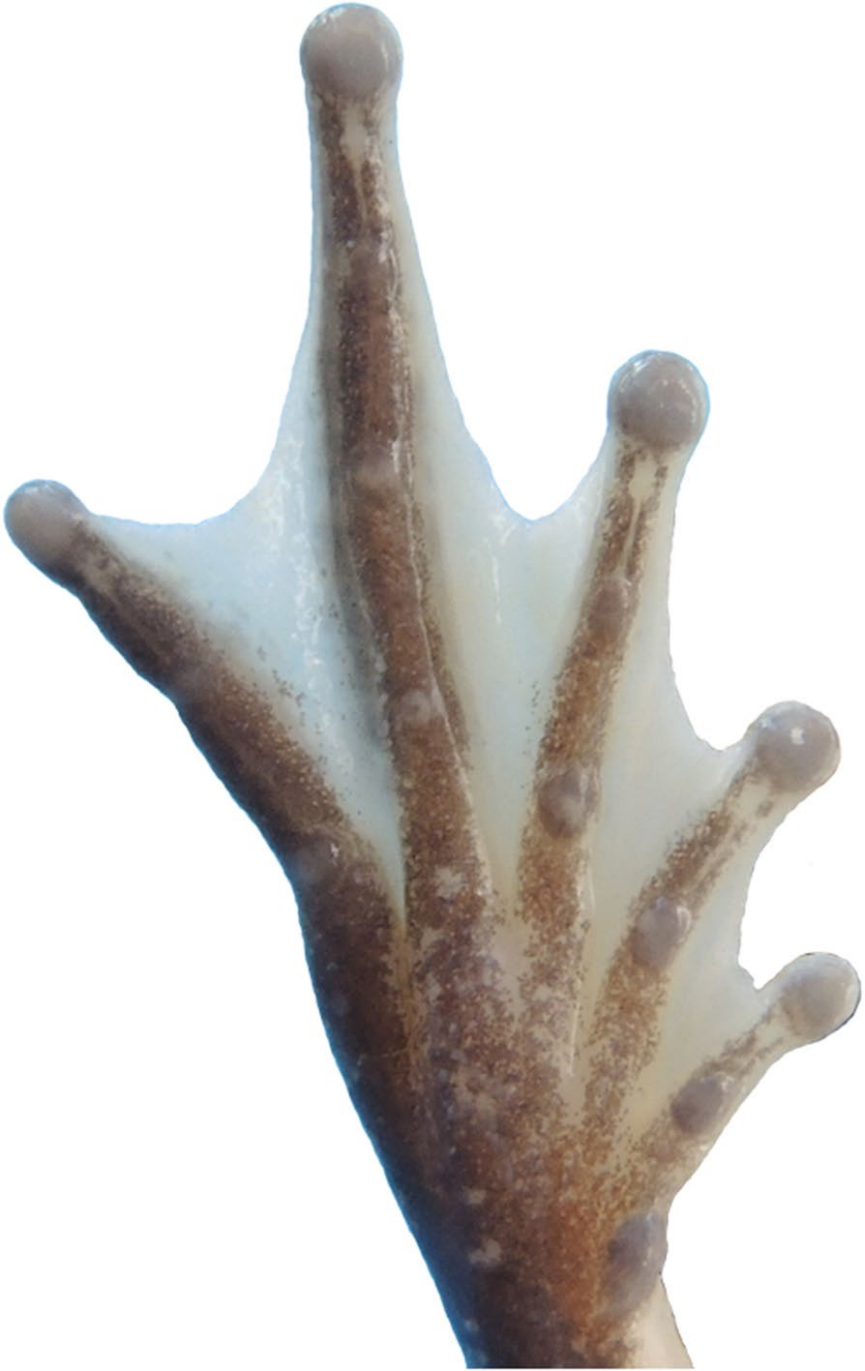
Ventral view of foot of the holotype of *Amolops ailao* sp. nov. in preservative

**Fig. 7:**
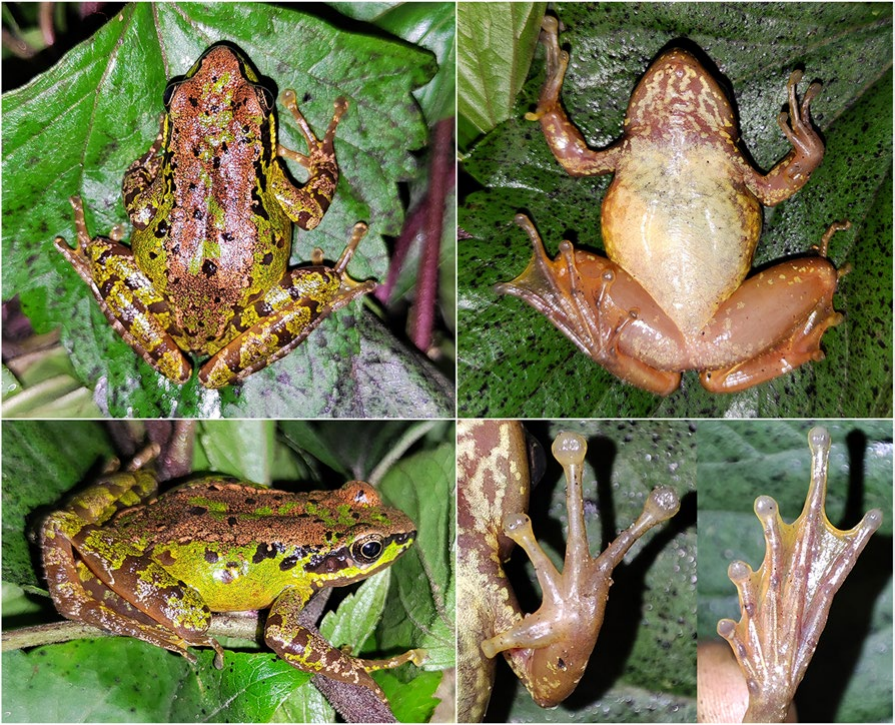
Views of the female paratype of *Amolops ailao* sp. nov. (KIZ 2022041) in life

**Fig. 8:**
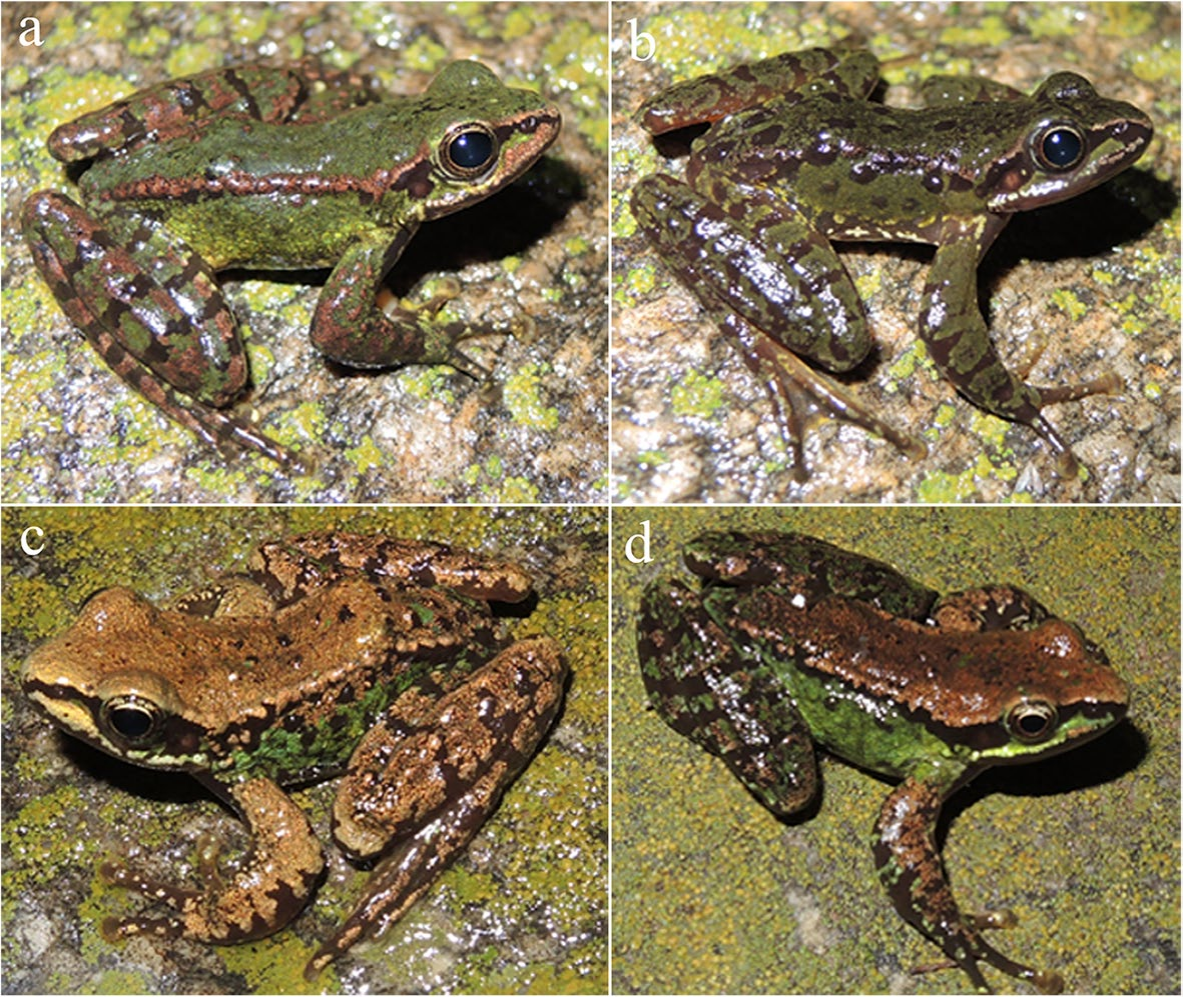
Dorsolateral views of male paratypes in life. (**a**) GXNU YU20160273; (**b**) GXNU YU20160274; (**c**) GXNU YU000003; and (**d**) GXNU YU000002

***Amolops ailao*** **sp. nov. (**Figs. 5, 6, 7 and 8; Table 4**)**

#### Zoobank

Urn:lsid:zoobank.org:act:725D8480-7921-46EF-975B-524EA75EF1A4.

#### Holotype

GXNU YU000004, an adult male, collected on 12 May 2019 by Guohua Yu from Mt. Ailao (23°56′58.20″N, 101°29′51.66″E, 2043 m above sea level; Fig. 1), Xinping County, Yunnan Province, China.

#### Paratypes

GXNU YU000001–YU000003, three adult males, collected at same time as the holotype from the type locality by Guohua Yu; GXNU YU20160273 and GXNU YU20160274, two adult males, collected from the type locality by Guohua Yu on 17 July 2017; and KIZ 2022041, an adult female, collected from the type locality by Shuo Liu on 22 June 2022.

#### Etymology

The specific epithet is named for the type locality, Ailao Mt., Xinping County, Yunnan Province, China. We suggest the English common name “Ailao cascade frog” and the Chinese common name “Āi Láo Tūan Wā (哀牢湍蛙)”.

#### Diagnosis

Morphologically, *Amolops ailao* sp. nov. resembles members of the *A. mantzorum* group in the absence of true dorsolateral folds and circummarginal groove on the disc of the first finger, and further resembles *A. jinjiangensis* and *A. shuichengicus* in the presence of folds formed by incomplete series of glands along the dorsolateral junction of the body (dorsolateral glandular folds). Phylogenetically, a clade consisting of the new species, *A. mantzorum*, *A. sangzhiensis*, *A. jinjiangensis*, *A. granulosus*, *A. loloensis*, *A. tuberodepressus*, *A. shuichengicus*, and *A. ottorum* was strongly supported (Clade I; Fig. 2). *Amolops ailao* sp. nov. can be distinguished from its congeners by the combination of the following characters: (1) body size small (SVL 33.0–35.1 mm in males and 41.3 mm in female); (2) HW/SVL 0.32‒0.35; UEW/SVL 0.08‒0.10; THL/SVL 0.52‒0.56; (3) vomerine teeth absent; (4) tympanum distinct, less than half eye diameter; (5) supratympanic fold present, indistinct; (6) true dorsolateral folds absent, but dorsolateral glandular folds distinct; (7) absence of circummarginal groove on the disc of the first finger; (8) tibiotarsal articulation reaching beyond anterior corner of eye; (9) dorsal surface smooth with no white spines; (10) a pair of large tubercles on sides of cloaca; 11) vocal sac absent; 12) toes fully webbed except the fourth; 13) interorbital space narrower than internarial space.

#### Description of holotype

Adult male (SVL 33.6 mm); head slightly longer (HL 11.0 mm) than wide (HW 10.8 mm); snout obtusely pointed, projecting beyond margin of lower jaw in ventral view, rounded in profile; canthus rostralis distinct, curved; loreal region sloping, concave; nostril oval, lateral, slightly protuberant; internarial distance (IND 3.7 mm) greater than interorbital distance (IOD 3.3 mm); upper eyelid width (UEW 3.0 mm) slightly narrower than interorbital distance; pineal spot present; pupil oval, horizontal; tympanum distinct, rounded, less than half eye diameter; supratympanic fold indistinct; vomerine teeth absent; choanae oval; tongue attached anteriorly, cordiform, notched posteriorly; vocal sac opening absent.

Forelimbs robust, relative length of fingers I < II < IV < III; tips of outer three fingers expanded into discs with circummarginal grooves, relative size of discs: 1 < 2 < 3 = 4; nuptial pads present on finger I; webbing between fingers absent; subarticular tubercles prominent and rounded, formula 1, 1, 2, 2; supernumerary tubercles present; thenar (inner metacarpal) tubercle oval; outer metacarpal tubercle single, rounded.

Hindlimbs long, heels overlapping when legs at right angle to body, tibiotarsal articulation reaching beyond anterior corner of eye; tibia length (TL 20.1 mm) longer than forearm and hand length (FHL17.6 mm), thigh length (THL 18.4 mm), and foot length (FL 19.8 mm); relative length of toes I < II < III < V < IV; all toe tips expanded into discs with circummarginal grooves; webbing between toes well developed, two third web, webbing formula I1–1.5II1–1.5III1–2IV2–1V; subarticular tubercles distinct, formula 1, 1, 2, 3, 2; inner metatarsal tubercle prominent, oval; outer metatarsal tubercle absent; supernumerary tubercles absent.

True dorsolateral folds absent, but folds formed by incomplete series of glands along dorsolateral junction of body (dorsolateral glandular folds) present, extending from rear of eye to groin; skin smooth, with a few flattened tubercles on flanks and dorsal surface of limbs; a few small tubercles on posterior surface of thigh and around vent; a pair of relatively large tubercles at the side of the anus; ventral surface smooth; a rictal gland.

#### Color of holotype

In life, iris light brown with dark wash; top of head and dorsum golden brown with large rounded black brown and green spots; sides of head with a pale green stripe extending from loreal region to region behind and below eye along upper lip; a short brown stripe below the green stripe on the loreal region; a black brown band from the tip of the snout through the nostril to an anterior border of the eye, continuing behind the eye to the shoulder; temporal region black brown with a green blotch; flanks green with few back brown and light yellow spots, a golden brown patch below dorsolateral glands; rictal gland pale green; limbs dorsally golden brown with black brown bands; anterior and posterior of forelimb and thigh black brown, mottled with green and light green blotches; throat, chest, and venter creamy white, mottled with light green and marbled with gray on throat and chest; venter of limbs flesh-colored, scattered with light green spots; web orange yellow.

In preservative, color faded. Dorsal surface light brown with beige brown and gray blue spots on head and body and beige brown bands on limbs; ventral surface white, marbled with brown on throat and chest.

#### Sexual dimorphism

Body size of males smaller than that of female; nuptial pads present on the base of finger I in males.

#### Morphological variation

Color of dorsal surfaces varied among specimens. Ground color of dorsal surfaces of the holotype and three paratypes (GXNU YU000002, GXNU YU000003, and KIZ2022041) is brown, and ground color of dorsal surfaces of remaining paratypes (GXNU YU20160273, GXNU YU20160274, and GXNU YU000001) is green. No large black brown spot on dorsum and flanks of GXNU YU20160273.

#### Distribution and ecology

**Fig. 9:**
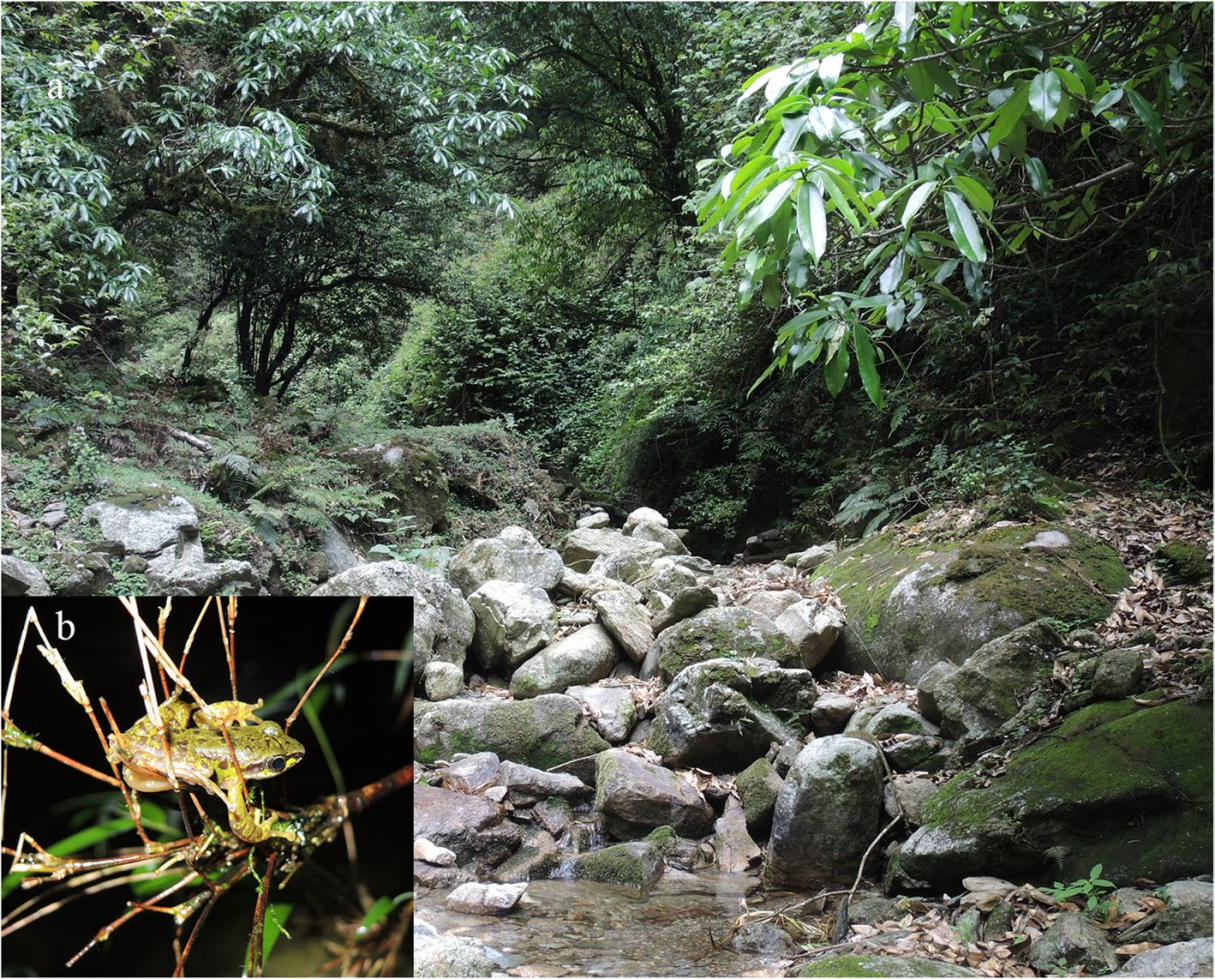
Habitat at the type locality of *Amolops ailao* sp. nov. (**a**) and an adult male of *Amolops ailao* sp. nov. sitting on branches at the type locality (**b**)

The new species is only known from the type locality. It was found on leaves or small branches less than 2 m above the ground along a mountain stream at night (Fig. 9) from May to July. All male types have nuptial pads on first finger and the female paratype (KIZ 2022041) is pregnant with eggs, suggesting that the breeding season may be from May to July. No tadpoles were collected for the new species. *Amolops tuberodepressus* was also encountered during surveys at the type locality.

#### Comparison

Within the *A. mantzorum* group, the new species can be distinguished from its sister taxon, *A. ottorum*, by smaller body size (female SVL 41.3 mm vs. 47.5‒48.2 mm in females), wider head (HW/SVL 0.32‒0.35 in the new species vs. HW/SVL 0.31 in *A. ottorum*), wider upper eyelid (UEW/SVL 0.08‒0.10 in the new species vs. 0.07 in *A. ottorum*), larger eye (ED/SVL 0.12‒0.14 vs. 0.12), and longer femur (THL/SVL 0.52‒0.56 vs. 0.49), the presence of dorsolateral glandular folds (versus absent), the presence of tubercles on flanks and limbs (versus absent), and the presence of a pair of large tubercles on sides of cloaca (versus absent). *Amolops tuberodepressus* also occurred at the type locality of the new species. *Amolops ailao* sp. nov. can be easily distinguished from *A. tuberodepressus* by smaller body size (males SVL 33.0–35.1 and female SVL 41.3 mm vs. males SVL 44.3–56.7 and females SVL 60.8–71.1 mm), vomerine teeth absent (vs. present), dorsolateral glandular folds present (versus absent), a pair of large tubercles on sides of cloaca (vs. absent), and tibiotarsal articulation reaching beyond anterior corner of eye (vs. tibiotarsal articulation reaching beyond tip of snout).

The new species is distinguishable from *A. minutus* by vomerine teeth absent (vs. strongly developed), vocal sac absent (vs. paired well-developed vocal sacs), and tympanum less than half eye diameter (vs. TD/ED mean 0.52 in males and mean 0.58 in females). The new species differs from the other seven members of the *A. mantzorum* species group (*A. sangzhiensis*, *A. shuichengicus*, *A. mantzorum*, *A. granulosus*, *A. jinjiangensis*, *A. lifanensis*, and *A. loloensis*) by vomerine teeth absent (vs. present) and smaller body size, males SVL 33.0–35.1 mm and female SVL 41.3 mm (vs. males SVL 40.3–40.9 mm and females SVL 52.6–57.7 mm in *A. sangzhiensis*, males SVL 34.6–39.6 mm and females SVL 48.5–55.5 mm in *A. shuichengicus*, males SVL 41.2–57.5 mm and females SVL 48.5–72.0 mm in *A. mantzorum*, males SVL 36.3–41.8 mm and female SVL 51.9 mm in *A. granulosus*, males SVL 43.0–52.0 mm and females SVL 54–66.4 mm in *A. jinjiangensis*, males SVL 52–56 mm and females SVL 61.0–79.0 in *A. lifanensis*, and males SVL 54.5–62.0 mm and females SVL 69.5–77.5 mm in *A. loloensis*).

*Amolops ailao* sp. nov. further differs from *A. mantzorum*, *A. lifanensis*, and *A. loloensis* by the presence of distinct dorsolateral glandular folds (vs. absent); from *A. mantzorum*, *A. jinjiangensis, A. lifanensis*, and *A. loloensis* by tympanum distinct (vs. obscure or invisible); and from *A. granulosus* by dorsal surface smooth with no white spines (vs. dorsal surface rough with spines) and vocal sac absent (vs. a pair of internal subgular vocal sacs).

*Amolops ailao* sp. nov. further differs from *A. jinjiangensis* by dorsal surfaces smooth (vs. skin coarse, with many rounded tubercles on head side, body side, and posterior part of dorsum); from *A. lifanensis* by toes fully webbed except the fourth (vs. webs fully developed to the bases of all toe disks); and from *A. loloensis* by interorbital space narrower than internarial space (vs. interorbital space about equal to the internarial space).

The *A. monticola* group contains 23 members, namely *A. adicola* Patel, Garg, Das, Stuart, and Biju, 2021 [12], *A. akhaorum* Stuart, Bain, Phimmachak, and Spence, 2010 [50], *A. aniqiaoensis* Dong, Rao, and Lü, 2005 [55], *A. archotaphus* (Inger and Chanard, 1997) [43], *A. bellulus* Liu, Yang, Ferraris, and Matsui, 2000 [74], *A. chakrataensis* Ray, 1992 [75], *A. chaochin* Jiang, Ren, Lyu, and Li, 2021 [11], *A. chunganensis* (Pope, 1929) [76], *A. compotrix* (Bain, Stuart, and Orlov, 2006) [38], *A. cucae* (Bain, Stuart, and Orlov, 2006) [38], *A. daorum* (Bain, Lathrop, Murphy, Orlov, and Ho, 2003) [37], *A. deng* Jiang, Wang, and Che, 2020 [34], *A. iriodes* (Bain and Nguyen, 2004) [39], *A. kohimaensis* Biju, Mahony, and Kamei, 2010 [40], *A. mengdingensis* Yu, Wu, and Yang, 2019 [22], *A. mengyangensis* Wu and Tian, 1995 [52], *A. monticola* (Anderson, 1871) [77], *A. nyingchiensis* Jiang, Wang, Xie, Jiang, and Che, 2016 [7], *A. putaoensis* Gan, Qin, Lwin, Li, Quan, Liu, and Yu, 2020 [41], *A. truongi* Pham, Pham, Ngo, Sung, Ziegler, and Le, 2023 [56], *A. tuanjieensis* Gan, Yu, and Wu, 2020 [18], *A. vitreus* (Bain, Stuart, and Orlov, 2006) [38], and *A. wenshanensis* Yuan, Jin, Li, Stuart, and Wu, 2018 [23]. *Amolops ailao* sp. nov. can be distinguished from these species by absence of true dorsolateral folds (vs. present). The new species can be further distinguished from *A. adicola*, *A. akhaorum*, *A. aniqiaoensis*, *A. archotaphus*, *A. chaochin*, *A. chunganensis*, *A. compotrix*, *A. cucae*, *A. daorum*, *A. iriodes*, *A. kohimaensis*, *A. mengdingensis*, *A. mengyangensis*, *A. monticola, A. putaoensis*, *A. truongi*, *A. tuanjieensis*, *A. vitreus*, and *A. wenshanensis* by vocal sac absent (vs. present); and from *A. adicola*, *A. akhaorum*, *A. aniqiaoensis*, *A. archotaphus*, *A. bellulus*, *A. chakrataensis*, *A. chaochin*, *A. chunganensis*, *A. compotrix*, *A. cucae*, *A. deng*, *A. iriodes*, *A. kohimaensis*, *A. mengdingensis*, *A. mengyangensis*, *A. nyingchiensis*, *A. putaoensis*, *A. truongi*, *A. tuanjieensis*, *A. vitreus*, and *A. wenshanensis* by vomerine teeth absent (vs. present).

*Amolops ailao* sp. nov. is distinguishable from *A. chayuensis* Sun, Luo, Sun, and Zhang, 2013 [21], the sole member of the *A. chayuensis* group, by true dorsolateral folds absent (vs. present), vocal sacs absent (vs. present), and vomerine teeth absent (vs. present).

The *A. viridimaculatus* group contains 14 species based on recent taxonomic studies [11, 13, 57], namely *A. beibengensis* Jiang, Li, Zou, Yan, and Che, 2020 [33], *A. chanakya* Saikia, Laskar, Dinesh, Shabnam, and Sinha, 2022 [57], *A. formosus* (Günther, 1876) [78], *A. himalayanus* (Boulenger, 1888) [79], *A. kaulbacki* (Smith, 1940) [80], *A. longimanus* (Andersson, 1939) [81], *A. medogensis* Li and Rao, 2005 [55], *A. nidorbellus* Biju, Mahony, and Kamei, 2010 [40], *A. pallasitatus* Qi, Zhou, Lyu, Lu, and Li, 2019 [2], *A. senchalensis* Chanda, 1987 “1986” [82], *A. tawang* Saikia, Laskar, Dinesh, Shabnam, and Sinha, 2022 [57], *A. wangyali* Mahony, Nidup, Streicher, Teeling, and Kamei, 2022 [13], *A. wangyufani* Jiang, 2020 [34], and *A. viridimaculatus* (Jiang, 1983) [45]. The new species can be distinguished from these species by vomerine teeth absent (vs. present), glands in compete series along dorsolateral junction present (vs. absent), and smaller body size (vs. male SVL 75.8 mm and females SVL 90.2–93.2 mm in *A. beibengensis*, male SVL 76.4 mm in *A. chanakya*, males SVL 61.3–63.1 mm and females SVL 79.4–83.7 mm in *A. formosus*, male SVL 80 mm in *A. himalayanus*, males SVL 70–72 mm in *A. kaulbacki*, male SVL ca. 95 mm and females SVL72.4–96.9 mm in *A. medogensis*, males SVL 76.4–82.3 mm and females SVL 85.4–98 mm in *A. nidorbellus*, female SVL 70.6–72.3 mm in *A. pallasitatus*, male SVL 46.2 mm in *A. senchalensis*, male SVL 82.5 mm in *A. tawang*, males SVL 71.4–76.7 mm and females SVL 80.5–89.6 mm in *A. wangyali*, males SVL 68.3–69.0 mm and female SVL 83.4 mm in *A. wangyufani*, males SVL 72.7–82.3 mm and female SVL 83.0–94.3 mm in *A. viridimaculatus*).

The *Amolops marmoratus* group contains 13 species, namely *A. afghanus* (Günther, 1858) [83], *A. assamensis* Sengupta, Hussain, Choudhury, Gogoi, Ahmed, and Choudhury, 2008 [49], *A. gerbillus* (Annandale, 1912) [84], *A. indoburmanensis* Dever, Fuiten, Konu, and Wilkinson, 2012 [6], *A. jaunsari* Ray, 1992 [75], *A. latopalmatus* (Boulenger, 1882) [85], *A. mahabharatensis* Khatiwada, Shu, Wang, Zhao, Xie, and Jiang, 2020 [10], *A. marmoratus* (Blyth, 1855) [86], *A. nepalicus* Yang, 1991 [53], *A. panhai* Matsui and Nabhitabhata, 2006 [47], *A. siju* Saikia, Sinha, Shabnam, and Dinesh, 2023 [59], *A. terraorchis* Saikia, Sinha, Laskar, Shabnam, and Dinesh, 2022 [58], and *A. yarlungzangbo* Jiang, Wang, Li, Qi, Li, and Che, 2020 [34]. The new species differs from these species by circummarginal groove on disc of finger I absent (vs. present), vomerine teeth absent (vs. present), and vocal sac absent (vs. present with the exception of *A. siju*).

The new species differs from *A. spinapectoralis* Inger, Orlov, and Darevsky, 1999 [41], the sole member of the *A. spinapectoralis* group, by vomerine teeth absent (vs. present), circummarginal groove on disc of finger I absent (vs. present), and vocal sac absent (vs. present).

The *A. larutensis* group contains four species, namely *A. australis* Chan, Abraham, Grismer, and Grismer, 2018 [8], *A. cremnobatus* Inger and Kottelat, 1998 [44], *A. gerutu* Chan, Abraham, Grismer, and Grismer, 2018 [8], and *A. larutensis* (Boulenger, 1899) [87]. The new species can be distinguished from these species by vomerine teeth absent (vs. present), circummarginal groove on disc of finger I absent (vs. present), and vocal sac absent (vs. present).

The *A. ricketti* group contains eight species, namely *A. shihaitaoi* Wang, Li, Du, Hou, and Yu, 2022 [5], *A. sinensis* Lyu, Wang, and Wang, 2019 [19], *A. ricketti* (Boulenger, 1899) [88], *A. wuyiensis* (Liu and Hu, 1975) [46], *A. yunkaiensis* Lyu, Wang, Liu, Zeng, and Wang, 2018 [9], *A. albispinus* Sung, Wang, and Wang, 2016 [51], *A. yatseni* Lyu, Wang, and Wang, 2019 [19], and *A. tonkinensis* (Ahl, 1927 “1926’’) [89]. The new species differs from these species by circummarginal groove on disc of finger I absent (vs. present), dorsolateral glandular folds present (vs. absent), and nuptial pad without conical or papillate nuptial spines (vs. present).

The *A. daiyunensis* group contains three species, namely *A. daiyunensis* (Liu and Hu, 1975) [46], *A. hongkongensis* (Pope and Romer, 1951) [90], and *A. teochew* Zeng, Wang, Lyu, and Wang, 2021 [54]. The new species differs from them by circummarginal groove on disc of finger I absent (vs. present), dorsolateral glandular folds present (vs. absent), and vocal sac absent (vs. present).

The *A. hainanensis* group contains two members, namely *A. hainanensis* (Boulenger, 1900) [91] and *A. torrentis* (Smith, 1923) [92]. The new species can be distinguished from them by dorsolateral glandular folds absent (vs. absent), circummarginal groove on disc of finger absent (vs. present), and nuptial pad present in males (vs. absent).

The new species is distinguishable from *A. binchachaensis* Rao, Hui, Ma, and Zhu, 2022 “2020” [35], which has not been assigned to any species group, by true dorsolateral folds absent (vs. present) and circummarginal groove on disc of finger I absent (vs. present).

## Discussion

Based on molecular and morphological evidence, we find a novel lineage belonging to the *A. mantzorum* species group from central Yunnan, China and describe it as a new species. This finding brings the number of species of the *A. mantzorum* group to 11. The phylogenetic relationships within the clade consisting of all members of the *A. mantzorum* group with the exception of *A. lifanensis* (labelled as clade I) were not well resolved and most basal branches in this clade are short (Fig. 2), suggesting that this group might have undergone a rapid speciation process. This inference was supported by the analysis of divergence dating, which revealed that the cladogenesis events within the *A. mantzorum* group mainly occurred from Pliocene 4.23 Mya (95% HPD: 2.56‒6.1 Mya) to Pleistocene 1.2 Mya (95% HPD: 0.53‒1.98 Mya) (Fig. 3). It is generally believed that the southeastern margin of the Qinghai-Tibet Plateau has experienced rapid and recent uplift since the Pliocene [93–95] and there were two phases of recent intense uplift occurring between 0.6 and 3.4 Mya [93], which caused dramatic habitat and climatic changes (e.g., reorganization of drainage [96] and formation of fluvial system [97]) and created environmental conditions (new habitats, dispersal barriers, etc.) that increase the rate at which species divide and evolve to form new ones [98]. Additionally, so far *A. minutus* has never been included in phylogenetic analysis. Thus, more studies are necessary to unveil the phylogenetic relationships within the *A. mantzorum* species group.

The phylogenetic position of *A. jinjiangensis* in previous studies is controversial. Lu et al. [63] found that *A. jinjiangensis* is closely related to *A. mantzorum*, but recently Lyu et al. [20] and Wang et al. [99] revealed that *A. jinjiangensis* is the sister taxon to *A. loloensis*. The samples of *A. jinjiangensis* in Lu et al. [63] came from Zhongdian in Yunnan and Yajiang in Sichuan, while samples of *A. jinjiangensis* in Lyu et al. [20] and Wang et al. [99] contained topotypes from Deqin, Yunnan. In this study, with inclusion of samples from both Zhongdian and the type locality (Deqin, Yunnan), we found that *A. jinjiangensis* contains two clades. The clade containing topotypes was recovered as the sister to *A. loloensis*, whereas the clade containing the sample from Zhongdian was nested in the clade of *A. mantzorum* (Fig. 2), indicating that the samples from Zhongdian in Lu et al. [63] actually do not belong to *A. jinjiangensis* but belong to *A. mantzorum*. *Amolops mantzorum* is comprised of four sub-lineages and the central lineage refers to *A. mantzorum mantzorum* according to Frost [14]. Thus, additional studies are needed to name the northern lineage and the lineage containing samples from Zhongdian in Yunnan.

**Fig. 10:**
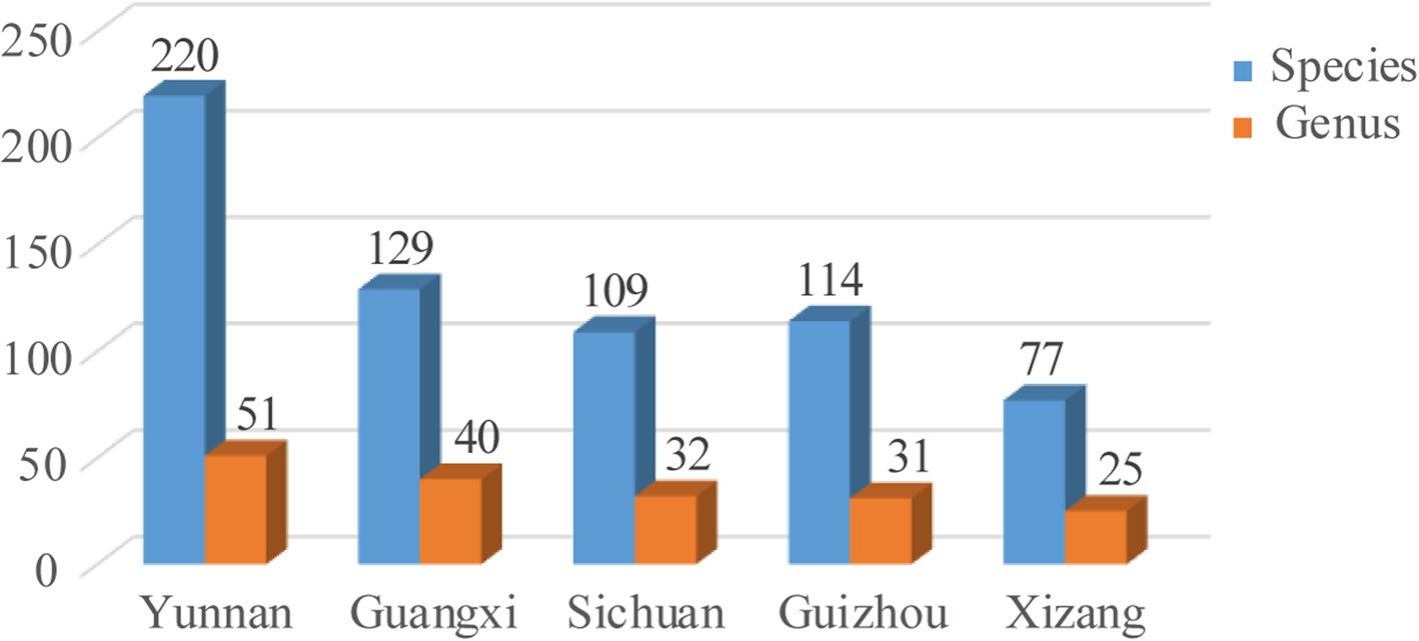
Comparison of amphibian diversity between Yunnan and adjacent provinces on species and genus level

**Fig. 11:**
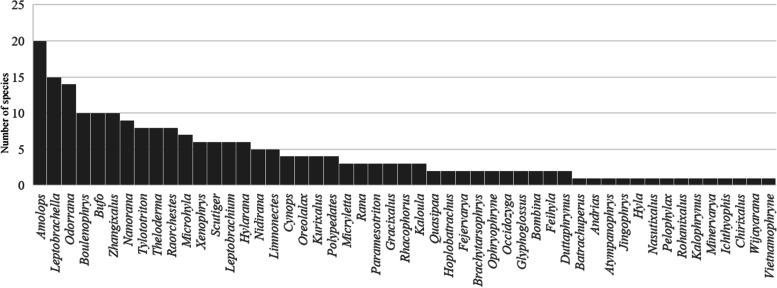
Contributions of *Amolops* and other genera to the amphibian diversity in Yunnan, China

**Fig. 12:**
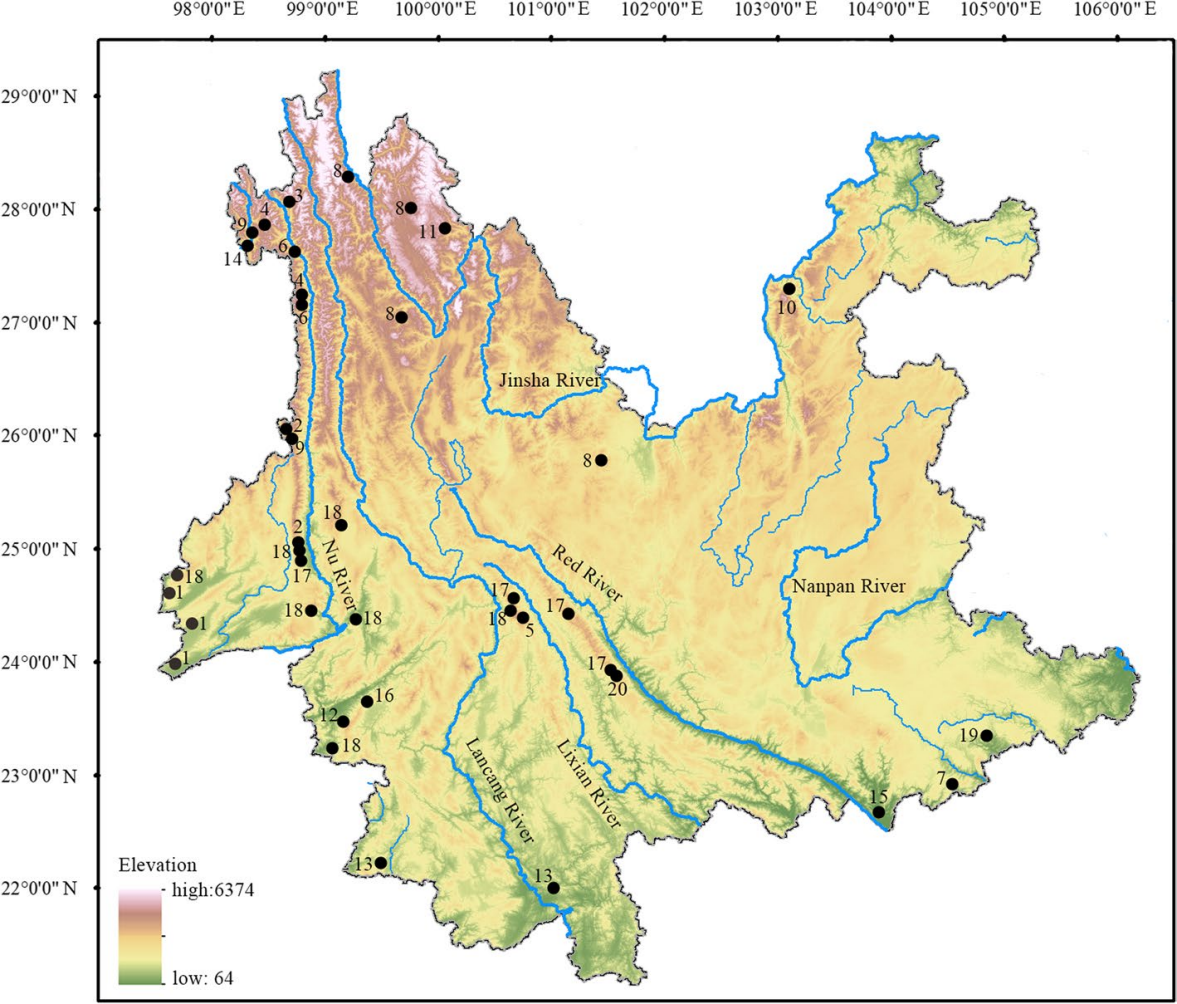
Geographic distribution of *Amolops* species in Yunnan, China. 1, *A. afghanus*; 2, *A. bellulus*; 3, A. *binchachaensis*; 4, *A. chayuensis*; 5, *A. daorum*; 6, *A. deng*; 7, *A. iriodes*; 8, *A. jinjiangensis*; 9, *A. kaulbacki*; 10, *A. loloensis*; 11, *A. mantzorum*; 12, *A. mengdingensis*; 13, *A. mengyangensis*; 14, *A. putaoensis*; 15, *A. shihaitaoi*; 16, *A. tuanjieensis*; 17, *A. tuberodepressus*; 18, *A. viridimaculatus*; 19, *A. wenshanensis*; 20, *A. ailao* sp. nov

Thanks to the extremely complicated topography and climatic condition in Yunnan, which promoted rapid divergence and speciation in small and isolated populations [100], Yunnan is the region with highest amphibian species diversity in China [15]. Of the 649 amphibian species known from China, about one-third (220 species belonging to 51 genus) are distributed in Yunnan (Fig. 10). Combining findings in this study with most recent taxonomic progress [5, 13, 35, 36], we consider that so far there are 20 *Amolops* species known from Yunnan, accounting for the highest proportion of amphibian diversity of Yunnan (ca. 9.1%; Fig. 11) and including five members of the *A. mantzorum* group (namely, *A. ailao* sp. nov., *A. jinjiangensis*, *A. loloensis*, *A. tuberodepressus*, and *A. mantzorum*), one member of the *A. marmoratus* group (namely *A. afghanus*), eight members of the *A. monticola* group (namely *A. bellulus*, *A. daorum*, *A. deng*, *A. iriodes*, *A. mengdingensis*, *A. mengyangensis*, *A. putaoensis*, *A. tuanjieensis*, and *A. wenshanensis*), two members of the *A. viridimaculatus* group (namely *A. viridimaculatus*, *A. kaulbacki*), one member of the *A. ricketti* group (namely *A. shihaitaoi*), one member of the *A. chayuensis* group (*A. chayuensis*), and *A. binchachaensis*, which has not yet been assigned to any species group but likely belongs to the *A. monticola* group owing to the fact that it has true dorsolateral folds and head side dark with white upper lip stripe. Among different parts of Yunnan, the species diversity of *Amolops* in northwestern Yunnan was the highest (eight species, namely *A. bellulus*, *A. binchachaensis*, *A. chayuensis*, *A. deng*, *A. jinjiangensis*, *A. kaubacki*, *A. mantzorum*, *A. putaoensis*), followed by western Yunnan (six species, namely *A. afghanus*, *A. bellus*, *A. mengdingensis*, *A. tuanjieensis*, *A. tuberodepressus*, and *A. viridimaculatus*), central Yunnan (four species, namely *A. daorum*, *A. tuberdepressus*, *A. viridimaculatus*, and *A. ailao* sp. nov.), and southeastern Yunnan (three species, namely *A. iriodes*, *A. shihaitaoi*, and *A. wenshanensis*) in order, while southern and northeastern Yunnan have only one species each (Fig. 12). Accordingly, species diversity of *Amolops* in the Nu River Basin is highest (seven species, namely *A. bellulus*, *A. binchachaensis*, *A. chayuensis*, *A. deng*, *A. kaulbacki*, *A. tuberodepressus*, and *A. viridimaculatus*), followed by the Lancang River Basin (six species, namely *A. daorum*, *A. mengdingensis*, *A. mengyangensis*, *A. tuanjieensis*, *A. tuberodepressus*, and *A. viridimaculatus*), the Red River Basin (five species, namely *A. iriodes*, *A. shihaitaoi*, *A. tuberodepressus*, *A. wenshanensis*, and *A. ailao* sp. nov.), the Dulong River Basin (three species, namely *A. chayuensis*, *A. kaulbacki*, and *A. putaoensis*) and the Jinsha River Basin (three species, namely *A. jinjiangensis*, *A. loloensis*, and *A. mantzorum*), while there are two species (*A. afghanus* and *A. viridimaculatus*) in the Basins of the Ying River and the Ruili River (Fig. 12), both of which flow to the Irrawaddy River. *Amolops ailao* sp. nov. is sympatric with *A. tuberodepressus* at the type locality, but it is easy to distinguish them because the new species has smaller body size and dorsolateral glandular folds and lacks vomerine teeth. *Amolops mantzorum* was widely recorded from central, southwestern, and northwestern Yunnan [15, 17, 101]. Certainly some of these records actually apply to *A. tuberodepressus* or *A. jinjiangensis* because they were once placed into the synonymy of *A. mantzorum* by Fei et al. [17, 101]. Studies based on additional sampling will be necessary to clarify the species boundary within the *A. mantzorum* species group in Yunnan, China.

## Conclusions

In summary, based on morphological and molecular evidence, we revealed a new cascade frog species belonging to the *A. mantzorum* species group. The new species *Amolops ailao* sp. nov. is only found in Mt. Ailao, central Yunnan, China and has considerable variation of color pattern. Including *A. ailao* sp. nov., now there are 20 *Amolops* species known from Yunnan, China and five of them belong to the *A. mantzorum* species group. We also revealed that the samples of *A. jinjiangensis* in Lu et al. [58] are misidentification of *A. mantzorum*. The *A. mantzorum* species group has undergone a rapid speciation process since the Pliocene, coinciding with the recent rapid uplift of the Qinghai-Tibetan Plateau since the Pliocene. Among different subfauna and water systems in Yunnan, northwestern Yunnan and Nu River Basin harbor the highest species diversity of *Amolops*. The findings in this study improve our understanding of the species diversity of the genus *Amolops*. More studies are necessary to unveil the phylogenetic relationships and species boundaries within the *A. mantzorum* species group.

## Data Availability

All data generated or analyzed during this study are included in this published article. Sequences are deposited in GenBank, NCBI.
